# Differential Gene Expression Analysis of Whole Blood Transcriptome Between Young and Old Border Collie Dogs

**DOI:** 10.3390/vetsci12020086

**Published:** 2025-01-23

**Authors:** Dávid Jónás, Kitti Tátrai, Zsófia Rékasi, Balázs Egyed, Eniko Kubinyi

**Affiliations:** 1 Department of Ethology, ELTE Eötvös Loránd University, Pázmány Péter sétány 1/c, 1117 Budapest, Hungary; jonas.david@ttk.elte.hu (D.J.);; 2 MTA-ELTE Lendület “Momentum” Companion Animal Research Group, Pázmány Péter sétány 1/c, 1117 Budapest, Hungary; 3 Department of Genetics, ELTE Eötvös Loránd University, Pázmány Péter sétány 1/c, 1117 Budapest, Hungary; 4 ELTE NAP Canine Brain Research Group, Pázmány Péter sétány 1/c, 1117 Budapest, Hungary

**Keywords:** RNA sequencing, dog, aging, differential gene expression analysis, whole blood

## Abstract

Aging is the most significant risk factor for many diseases and increased mortality, and it is influenced by both genetic and environmental factors. In this study, our primary goal was to investigate age-related gene expression changes in whole blood samples collected from dogs and identify potential biomarkers of healthy aging. We sequenced the mRNA fraction of whole blood samples from five young and five old border collie dogs and performed differential gene expression and differential transcript usage analyses. The raw sequencing data exhibited high quality. Multidimensional scaling analysis failed to differentiate age clusters. Moreover, we identified only a limited number of differentially expressed genes (n = 61) and 30 genes with differential transcript usage between the blood transcriptomes of young and old dogs. Our results align with publicly available data on dogs. However, studies on other species, such as wolves, have identified more significant age-related genes. In conclusion, while some of our findings are promising, further research is needed to standardize environmental factors affecting blood gene expression levels in dogs. Additionally, we recommend implementing pre-sequencing hemoglobin depletion to improve the analysis of whole blood in future studies.

## Simple Summary

Aging is a major risk factor for various diseases. Understanding the mechanisms of healthy aging can aid in developing treatments for elderly individuals and promoting healthier lifestyles. Studying aging in humans is challenging due to their long lifespans, making companion dogs valuable alternative models because of their shared environment with humans and shorter lifespans. This study analyzed gene expression differences in whole blood between young adults and elderly healthy border collie dogs to identify potential biomarkers of healthy aging. Whole blood is a practical and minimally invasive sample source, reflecting systemic physiological changes and enabling longitudinal studies. The study found only a limited number of genes with significant age-related differences, which aligns with findings in dogs but contrasts with studies in other species that report broader transcriptomic changes with age. These results underscore the need for further research to clarify the blood transcriptomic basis of healthy aging in dogs and humans.

## 1. Introduction

Aging is one of the most significant risk factors for illnesses and mortality [1]. A wide range of age-related changes (e.g., hearing loss) and neurodegenerative disorders can severely reduce the quality of life of older people. Aging is influenced by environmental and genetic factors [2], but gene–environment interactions also play an essential role in aging-related phenotypes [3]. These biological changes are further compounded by the aging populations globally [4]. Companion animals face similar challenges in their older ages, and changes in age structures have emerged due to an ever-increasing quality of veterinarian care and likewise due to the changing owner care towards companion animals in the past decades [5].

Although lifespans differ significantly among species, improved healthcare in both human and veterinarian medicine has enabled humans and companion animals to live longer, closer to their species’ maximum lifespan. In dogs, however, significant within-species variance exists due to differences in lifespan among breeds and sizes (e.g., [6]). Consequently, it is of great importance not only for improving quality of life but also for addressing the associated social and economic challenges in humans and in agriculturally important or companion species.

A model organism with a much shorter lifespan than humans provides opportunities to study all factors influencing aging, including both environmental and genetic components. In recent years, several authors have recommended the dog as a model for human aging (e.g., [7,8]) and for studying age-related diseases, such as Alzheimer’s disease in humans [9]. This recommendation is supported by many characteristics of the domestic dog that make it an ideal model species for aging: companion dogs share a very similar environment with their owners, and they naturally develop some age-related diseases that are analogous to human diseases (e.g., [10]). Dogs also have a considerably shorter lifespan than their owners, making longitudinal studies easier. For a detailed review of the relevance of dogs in the study of the genetic background of human aging, see [11].

A previous study investigated the differential gene expression profile in the prefrontal cortex of young and old dogs to characterize gene expression changes associated with healthy aging [12]. While that study focused on cognitive aging and age-related gene expression changes, it was less practical for identifying genetic biomarkers of aging, as a pre-mortem collection of brain tissue samples is not feasible. In the current study, we extend this research by examining the same parameters in a more accessible tissue—whole blood. Unlike the brain tissue used in our previous study, which was obtained through the Canine Brain and Tissue Bank [13], whole blood offers a significant advantage as it can be easily collected from living subjects. Consequently, blood-based biomarkers of aging or genetic disorders hold greater practical value for both animal and human healthcare. For this reason, we decided to sequence whole blood samples.

However, using blood tissue for gene expression analysis has certain disadvantages. Whole blood contains a large number of red blood cells, which may contribute considerable amounts of hemoglobin (Hg)-related mRNA [14]. These hemoglobin-derived mRNA molecules can constitute a significant proportion of the total mRNA in an RNA sequencing experiment. As a result, other mRNA molecules may be underrepresented unless the sequencing depth is substantially increased, similar to RNA-seq experiments where ribosome depletion is not performed beforehand. Harrington et al. [14] demonstrated that implementing globin RNA depletion prior to RNA sequencing of whole blood is beneficial in blood transcriptome studies.

In our study, instead of employing pre-sequencing hemoglobin removal, we opted for in silico hemoglobin depletion. This approach allowed us to analyze whole blood transcriptomes and identify aging biomarkers without altering the natural composition of the samples. Moreover, the two publicly available blood transcriptome datasets from dogs [15,16] also lack pre-sequencing hemoglobin depletion, ensuring consistency with existing studies. A more recent study has introduced RNA library preparation protocols for hemoglobin-rich tissues in dogs, which include a globin depletion step [17].

Whole blood has been extensively studied over decades in connection with a wide range of diseases and phenotypes. Several blood biomarkers for specific diseases have already been identified. For example, potential biomarkers for the early diagnosis of Alzheimer’s disease have been reported using whole blood RNA sequencing data [18], and similar approaches have been applied to Parkinson’s disease [19]. However, while significant progress has been made in identifying genetic markers for specific diseases, the gene expression changes associated with healthy aging remain less explored.

Age-related changes in RNA levels have been studied in humans, both in lymphocytes [20] and whole blood [21,22]. Peters et al. [21] conducted a large-scale study that identified approximately 1500 differentially expressed genes associated with age. They found that these genes were often linked to active CpG methylation sites, and their associated pathways included transcription and translation dysregulation, DNA damage accumulation, immune senescence, and ribosome biogenesis. Additionally, novel pathways, such as actin remodeling, were discovered, highlighting previously unexplored connections to aging. Similarly, Viñuela et al. [22], in a twin study (age range: 39–85 years), identified 680 protein-coding genes with at least one exon exhibiting age-associated changes in expression. They reported that age explained approximately 5% of the variance in gene expression for these exons.

In wild gray wolves, Charruau et al. [23] analyzed blood gene expression patterns and identified 625 age-associated genes linked to processes such as immune response and RNA metabolism. In domesticated dogs, a recent study from 2023 reported 154 genes significantly associated with aging [24]. However, the absence of multiple testing corrections in this study raises concerns about the validity of the results. Notably, none of the reported genes would remain significant if an adjusted *p*-value had been applied. Zeng et al. [25] also investigated the age-related transcriptomic changes in dogs’ blood and identified 61 differentially expressed genes related to metabolism and the immune system.

The primary aim of our study was to investigate gene expression differences at the RNA level between two distinct age cohorts of dogs using whole blood samples and to identify potential biomarkers of healthy aging in adulthood. Similar to previous studies [18], we aimed to identify blood-based biomarkers associated with healthy aging.

To focus exclusively on age-related changes in RNA expression levels during adulthood, we did not include puppies in the analysis, thereby avoiding the confounding effects of developmental and adolescence-related changes in gene expression. Additionally, we selected a single, well-studied breed for this analysis to minimize the potential confounding effects of between-breed variability in both aging-related gene expression patterns and overall blood transcriptomes. This approach also allowed us to eliminate the influence of the well-documented negative correlation between body size and lifespan in dogs, thus avoiding the need for breed-specific lifespan corrections or the estimation of relative ages. We selected the border collie breed for this study due to its popularity and availability in sufficient numbers across both age cohorts. By focusing on two distinct age groups instead of treating age as a continuous variable, we aimed to simplify the model and increase statistical power, enabling the detection of significant age-related differences with a smaller sample size. These experimental design decisions were implemented to reduce or eliminate confounding factors, ensuring that the available statistical power was fully utilized to detect changes specifically associated with healthy aging.

## 2. Materials and Methods

### 2.1. Ethics

All procedures were conducted in compliance with national and EU legislation, as well as institutional guidelines, and adhered strictly to the recommendations of the International Society for Applied Ethology regarding the use of animals in research. Additionally, all methods followed the ARRIVE guidelines. The study was ethically approved by the Hungarian Pest County Governmental Office (Permission No.: PE/EA/301-4/2021). Written informed consent was obtained from all participating dog owners, who were provided with comprehensive information about the study. Special care was taken to ensure that participants fully understood the consent process. The consent form detailed the identity of the researchers, the study’s aim, procedures, location, expected time commitment, personal and research data handling, and plans for data reuse. Participants were explicitly informed of their right to withdraw consent at any time.

### 2.2. Samples

Ten border collies were included in this experiment, which were divided into two distinct age groups. The young cohort comprised dogs aged 1–3 years (n = 5; mean age: 1.4 years), while the old cohort included dogs aged 10–15 years (n = 5; mean age: 12 years). All dogs were raised and kept as companion animals in Hungary and were unrelated. The breed and age of each dog were verified through official documents signed by their veterinarians. Owners were interviewed regarding their dogs’ health status, confirming that none of the animals exhibited any signs of illness or had taken medication within two weeks prior to sampling. Additionally, one milliliter of blood from each dog was sent to a veterinary diagnostic laboratory for independent health verification, which confirmed that all dogs were healthy (Table S1). Both cohorts included a mix of sexes and neuter statuses (Table 1).

### 2.3. Blood Sampling, RNA Extraction, Library Preparation and Sequencing

Three milliliters of blood was taken from the vena cephalica or the vena saphena lateralis in accordance with the principles of lege artis by an experienced veterinarian in the presence of the dog’s owner. In order to preserve the RNA fraction of the blood as intact as possible, it was collected directly into DNA/RNABlood Collection Tubes (Zymo Research, Irvine, CA, USA). Blood was gently mixed with the special RNA preservative fluid and stored at −20 °C. Quick-DNA/RNA Blood Tube kits (Zymo Research) were used for RNA extraction. Isolated RNA was stored in a −80 °C ultra-low temperature freezer until further processing.

RNA samples were treated with DNase to remove DNA contamination from the RNA samples. Protein coding RNA was purified via poly(A) capture, and library preparation was performed with the TruSeq^®^ Stranded mRNA Library preparation kit (Illumina, San Diego, CA, USA). Sample quality control was implemented after the DNase treatment and after the library preparation alike (RIN: 8.5–10.0 in all samples, indicating high-quality raw material). Following the successful library preparation, samples were sequenced at the iBioScience company (Pécs, Hungary) with a Novaseq 6000 Illumina sequencer machine. The minimum read number was set to 42 million paired end reads per sample, and the read length was 150 base pairs (bp). Sequence data were shared with us by the company in standard fastq format, and our data analysis started with the quality control of the raw sequence data.

In accordance with the recommendations of Harrington et al. [14], we performed an in silico hemoglobin RNA depletion, which is expected to improve the overall performance of a whole blood RNA-seq experiment in terms of sensitive measurement of gene expression levels in the samples.

### 2.4. Data Analysis

Throughout the analysis, we aimed to use the default parameters of each software. However, this was not always possible. In the following description, we specify every parameter setting (except the obligatory ones) for every software that we have changed. We also provide a short reasoning whenever our parameter setting was not straightforward.

A power analysis was carried out as implemented in the RnaSeqSampleSize R package [26]. The FDR level was set at 10%. Its default parameter value in the DESeq2 v. 1.24.0 R package was used for the differential gene expression analysis here, and the minimum fold change (rho) was set to 3. The estimated power was 0.98, indicating that the group sizes of five samples, combined with the high sequencing depth, were sufficient for the study.

### 2.5. Quality Check and Data Preparation

The FastQC software v. 0.11.7 ([27]; RRID:SCR_014583) with its default parameter settings was used to check the raw read quality. The -o option was set to change the output directory, and the --noextract and the -f option to define the file format. Following the quality control, the cutadapt software v. 1.18 ([28]; RRID:SCR_011841) was used to perform the following tasks: (1) remove the trailing high-quality guanine (G) bases from the 3′ end of the sequences, which were added to the shorter fragments by the Novaseq 6000 sequencer, a 2-dye sequencing system and (2) remove the adapter sequences. In addition to the -a and -A options to specify the 3′ and 5′ adapter sequences, we also used the -j option to increase the number of CPUs to be used to five; the -m option to discard all reads shorter than 50 base pairs and keep only the longer ones; the --nextseq-trim 20 option was used to remove the trailing G bases from the sequences. In a second cutadapt run, the -u 17 and -U 17 options were used to hard-trim some additional bases from the sequences that did not pass the FastQC quality control.

In the next step, we aligned the reads to the dog reference genome (genome version: CanFam 3.1, [29]; genome annotation: Ensembl v98, [30]). We used the HISAT2 split-read aligner v. 2.2.1 ([31]; RRID:SCR_015530) with the following options: -p 6; --dta; input files were provided with the -1 and -2 options, which allowed the processing of paired-end reads.

Next, we determined the dog homologs of the human hemoglobin genes published by Harrington et al. [14] and removed the reads aligned with the dog homologs of the hemoglobin genes for one part of the analysis (Table 2). The homologs were identified using in-house scripts. The reads were removed using the BEDtools v.2.25.0 (intersectBed; [32]; RRID:SCR_006646) and the Picard software packages v.2.18.23 (FilterSamReads tool; [33]; RRID:SCR_006525). The genes’ relevance in our samples was also investigated based on their actual expression levels. Although the hemoglobin genes were excluded from the differential gene expression analysis, the effect of their presence in the RNA library was investigated and discussed below. Hemoglobin reads were removed immediately after the alignment, directly from the bam files.

### 2.6. Performing Differential Gene Expression Analysis

Differential gene expression analysis was implemented between the young and old cohorts. The DESeq2 R package v. 1.24.0 ([34]; RRID: SCR_015687) was used to perform the differential gene expression analysis, as described in the workflow of Love et al. [35]. The input of the DESeq2 software is a count matrix, where ki,j is the number of aligned reads at gene i in animal j. This count table was generated with the featureCounts() function of the Rsubread v. 1.34.7 R package, as recommended in the cited workflow. The following options were used to generate the appropriate data table: isGTFAnnotationFile = T, isPairedEnd = T, countMultiMappingReads = F, the latter being an important prerequisite of the software [34].

We decided to apply the more conservative definition of expressed genes from the workflow, and therefore, in this study, all genes with at least ten reads in five or more samples were considered as an expressed gene in the blood. The minimum number of individuals (i.e., five) was selected as this was the size of both animal cohorts in our study. We applied the regularized logarithm transformation (rlog), which is recommended for our sample size. This choice was also supported by the transformation efficiency comparison of the three implemented transformations in the DESeq2 package (rlog, variance stabilizing transformation—or vst—and the log2 transformation; Figure S1). This transformation was used when the data were analyzed in the descriptive statistical tests. However, it was not used for the differential gene expression analysis.

No parameter other than chronological age was expected to systematically affect gene expression levels or the differential gene expression analysis. Therefore, the model used for the differential gene expression analysis included only an intercept and the age effect. False discovery rate (FDR)-adjusted *p*-values were calculated, with a default cutoff value of 0.1 applied to identify significantly differentially expressed genes. The analysis was performed both with and without excluding hemoglobin-related reads, enabling a comparison of the impact of hemoglobin genes on the dataset. Figures were created using R packages (ggplot2 (RRID:SCR_014601), DESeq2, Venn Diagram (RRID:SCR_002414)), while tables were primarily created in MS Office Excel. The outline of the analysis pipeline is shown in Figure S2.

### 2.7. Gene Ontology Analysis

The differential gene expression analysis was continued with a gene ontology (GO)—overrepresentation test of the significant genes compared to the background of all expressed genes detected in the canine blood (n = 12,966). This analysis was implemented using the pantherDB online tool v. 16 [36,37].

### 2.8. Calculating Differential Transcript Usage

Gene expression quantification was implemented using the Salmon software v. 1.10.3 [38]; RRID: RRID:SCR_017036), which was followed by the differential transcript usage analysis using the DRIMSeq v. 1.30.0 R package [39]. For Salmon, the default parameters and the analysis pipeline recommended by the authors on the software’s website were used. For the DRIMSeq package, the following parameters were changed from their default values to fit our dataset: minimum number of samples where genes should be expressed was changed to 10; minimum number of samples where features (i.e., transcripts) should be expressed was set to the sample size of the smallest group (5 in our case), and both minimal gene and feature expression levels were set to 10 as well. These changes were in accordance with the recommendations of the package’s authors, available at the R package’s Bioconductor site.

### 2.9. Additional Analyses

We observed a high proportion of secondary alignments in our data. These were unexpected in an RNA sequencing experiment, and therefore, we decided to investigate this phenomenon as well.

## 3. Results

### 3.1. Raw Data Quality

Table 3 presents the raw data statistics of the samples. While the young animals had a higher average sequencing depth (142 million vs. 128 million reads), this was primarily driven by an outlier sample (CL_y3), which was sequenced to 211 million reads. Excluding this individual, there was no significant difference in sequencing depth between the two groups. After removing the outlier, the only significant difference between the age groups was observed in the number of secondary alignments, which included hemoglobin genes, with the old cohort showing 11 million more reads (or 8% higher) in this category.

Although the raw data quality checks (including adapter trimming, removal of the high-quality poly-G sequences from the 3′ end of the reads, hard trimming of the ends of the sequences, and removal of reads shorter than 50 bp) removed 24% of the reads, approximately 100 million reads were still retained for the analysis per sample (ranging from 76 to 132 million reads in the young cohort and 91 to 103 million reads in the old cohort), corresponding to 50 million fragments.

The alignment rate was high (86%; on average 92 and 85 million reads aligned in the young and old cohorts, respectively). Therefore, our dataset was appropriate for the planned differential gene expression analysis.

We also investigated the hemoglobin genes and the number of reads that aligned with these genes. On average, 43 million reads (or 48% of the reads kept after raw data filtering) aligned to the hemoglobin genes. A large variation could be observed in the data with respect to the number of hemoglobin-related reads, ranging from 24 to 75% of the filtered reads in the samples.

The hemoglobin-related reads filtering led to a large and significant reduction in the total read number, which was reduced to 23–74 million for the different samples. This affected the samples differently, with the largest changes in the young cohort: three samples had 23–30 million reads, while two samples had 66 and 74 million reads after removing the hemoglobin-related reads. The range of the read numbers in the old cohort remained more similar, but considerable variation existed in that group as well (32–58 million reads per sample). The varying amounts of hemoglobin-related mRNA introduced an unwanted bias to our experiment.

The number of secondary alignments was also affected by the hemoglobin genes. The average proportion of secondary alignments compared to the primary alignments was 69%, but it went up to as high as 127%, i.e., more secondary alignments were present in some samples than primary alignments. However, when the hemoglobin reads were removed, the proportion of multi-mapped reads dropped to a normal level, and the secondary alignments were at an average level of 3.6% across the samples, with a negligible difference between the age cohorts (3.4% and 3.7% in the young and old cohorts).

Consequently, the hemoglobin reads and the associated genes—primarily due to the large within-cohort variation—represented a large, random bias in our dataset. As a result, as well as following the recommendations of Harrington et al. [14], both the hemoglobin genes and the associated reads were excluded from the downstream analysis. A reduction in statistical power is expected due to the large reduction in the read counts.

### 3.2. Descriptive Statistical Analysis

**Figure 1:**
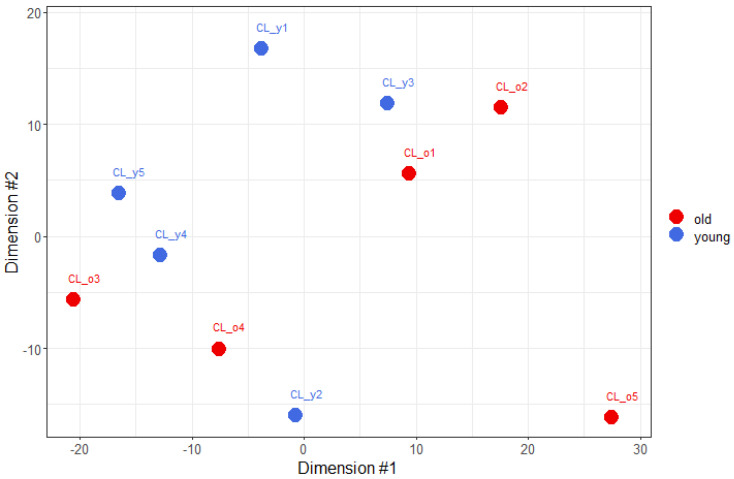
Multidimensional scaling plot of the read counts. Read counts were regularized logarithm transformed. The texts next to the dots represent the Sample ID-s, as shown in Table 3.

A total of 12,966 genes were expressed in the blood of the subjects. The age clusters could not be differentiated in a multidimensional scaling analysis, which was applied to the rlog-transformed read counts of the expressed genes (Figure 1). This suggests that the chronological age of dogs was not the primary source of the observed read count variance in our data. The two apparent clusters in Figure 1 were not related to any other known parameter (sex, neutered status, RNA concentration levels). However, they showed some relationship with the number of hemoglobin reads in the samples, with the bottom-left cluster having fewer hemoglobin-related reads (Figure 1, Table 3). This is most likely a random association, as hemoglobin reads were not included in the data when the figure was created.

Both a principal component analysis (Figure S3) and the Euclidean distances calculated from the rlog-transformed read counts of the samples (Figure S4) led to very similar observations, and neither of these two additional analyses could successfully differentiate the age groups. Thus, these analyses support the multidimensional scaling, that age was not the primary source of variation observed in the per-gene read counts.

### 3.3. Differential Gene Expression Analysis

**Figure 2:**
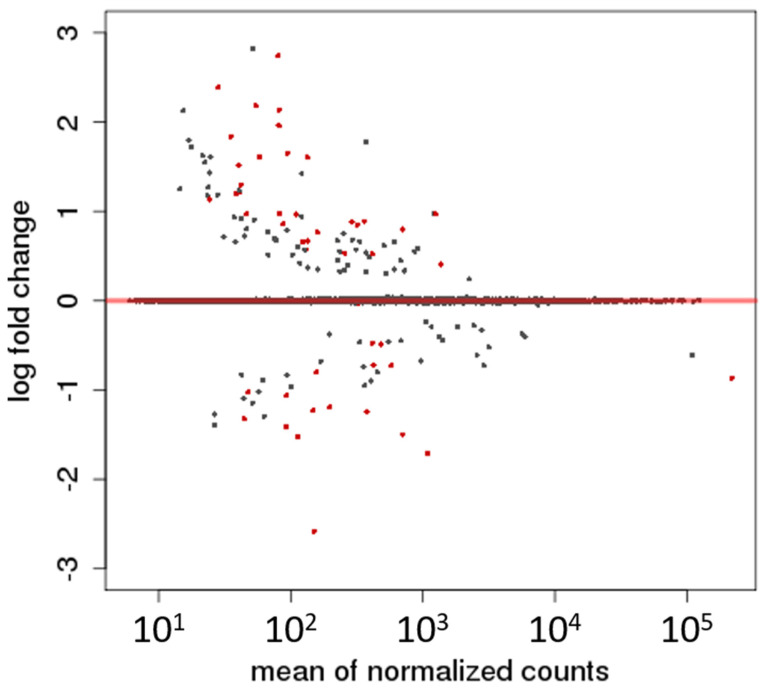
MA plot indicating the read counts and fold changes of all expressed genes in the samples. The differentially expressed genes are shown in red. Black dots are the insignificant genes. The red line indicates a 0-fold change.

Figure 2 shows an MA plot (where M refers to the fold change on the *y*-axis and A to the average expression level on the *x*-axis) of all expressed genes in the companion dogs’ blood tissue. The overwhelming majority of the genes had a log-fold change around 0: the fold change was between −1 and 1 for 12,541 (or 97%) of the expressed genes. This implies that gene expression changes in the blood transcriptome of the dogs as a function of age are exceptionally rare. This is true despite the significant differences between the number of aligned reads without the hemoglobin reads in the two age cohorts (Table 3).

**Figure 3:**
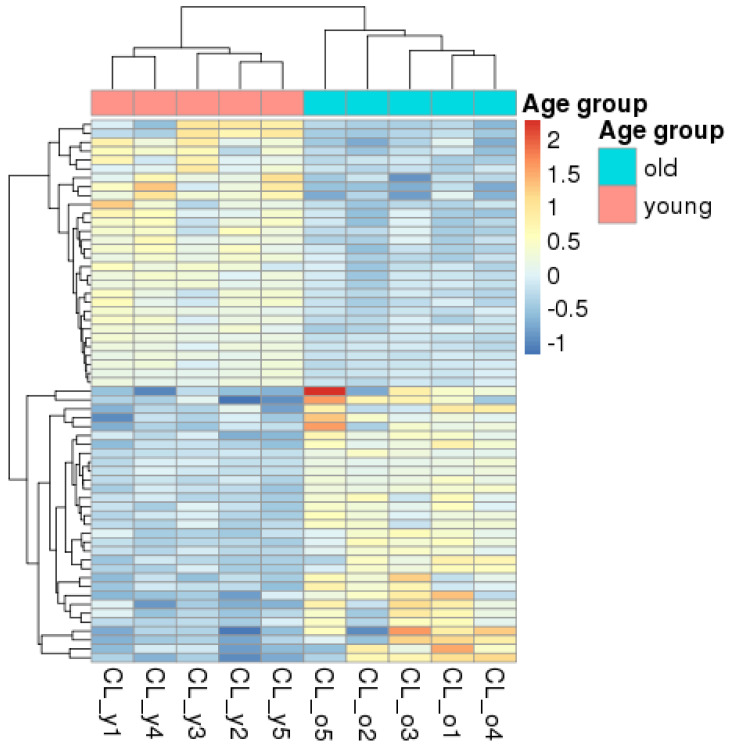
Heatmap showing the differentially expressed genes and the hierarchical clustering of both the genes (*x*-axis) and the individuals (*y*-axis).

We identified 61 differentially expressed genes, representing just 0.5% of all expressed genes. Of these, 31 were downregulated, and 30 were upregulated in old dogs compared to young dogs (Figure 3). The fold change in the significant genes ranged from 0.5 to 5.6. Clustering the sequenced animals based on the gene expression profiles of these differentially expressed genes clearly separated the two examined age groups, in contrast to the earlier analyses (e.g., MDS; Figure 1), which used normalized read counts of all expressed genes. (List of all differentially expressed genes together with additional information—such as gene biotype and gene version—from the reference annotation are available in Table S2). An identical differential gene expression analysis on both sex and neuter status resulted in no differentially expressed genes.

We also tested the effects of sex and neutering as covariates in the fitted model when age was the response variable, finding that neither parameter had a significant impact on the results. Using the same thresholds, only three out of the 12,966 tested genes were significantly differentially expressed between males and females, and none were differentially expressed between neutered and non-neutered animals.

Additionally, an independent, parallel differential gene expression analysis conducted with the edgeR R package, using its default parameter values, did not identify any differentially expressed genes between the two age groups.

### 3.4. Functional Analysis of the Differentially Expressed Genes

In the functional analysis of the differentially expressed genes, we found only one significantly enriched gene ontology term. The fold enrichment of “response to bacterium (GO:0009617)” was 8, and the corresponding false discovery rate adjusted *p*-value was 0.014.

### 3.5. Differential Transcript Usage

**Figure 4:**
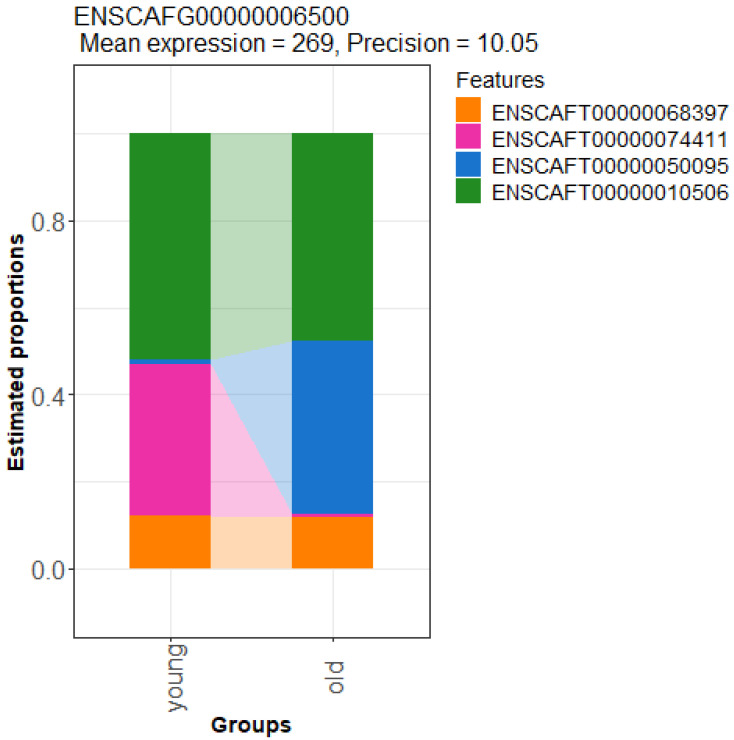
A ribbon plot of the TCERG1 gene with the largest differential transcript usage between the young and old age cohorts. Different colors correspond to the different transcripts of the gene.

A total of 32,739 transcripts across 16,666 genes were detected using Salmon, with an average of 1.9 transcripts per gene. The differential transcript usage analysis identified a very limited number of genes (n = 30) with significant shifts in transcript usage between the young and old cohorts (see example in Figure 4). This number is comparable to the range of differentially expressed genes identified.

### 3.6. Correlation Between RNA Concentrations and the Raw Number of Sequenced Reads

Unexpectedly, we found a strong correlation (r = 0.81) between the initial RNA concentrations and the raw number of sequenced fragments. For more details, see Supplementary Materials (Figure S5).

## 4. Discussion

Aging is a major factor in disease development and mortality. A recent study identified genes with significantly different expression levels between young and old dogs in the prefrontal cortex [12]. However, collecting brain tissue samples is highly challenging, making it impractical to monitor gene expression changes associated with pathological aging or disease. This limitation can be addressed by using whole blood, an easily accessible tissue, and a well-established source of biomarkers for various diseases [18,19]. In this study, we analyzed whole blood RNA sequencing data from healthy aging adult dogs to characterize genetic regulatory networks and identify potential biomarkers of healthy aging.

Interestingly, we observed a strong correlation between RNA concentration and the number of sequenced fragments. One plausible explanation is that higher RNA concentrations may indicate better RNA quality overall (i.e., intact, non-degraded RNA molecules), similar to observations in DNA PCR-based experiments. Experimental steps such as PCR reactions could have been more efficient with intact RNA, resulting in better sample and library quality, ultimately leading to higher sequencing efficiency and depth. Another potential explanation could be that samples with higher RNA concentration contained a greater number of cells or cell types, thereby increasing the complexity and amount of RNA available for sequencing. Although these factors are intriguing and warrant further investigation, they did not affect the analyses in this study, as both RNA quality and sequencing depth were sufficiently high, even in samples with the lowest coverage.

The produced raw data was of good quality. Although 24% of the reads were filtered out before alignment to the reference genome, due to the very high initial sequencing depth, more than 100 million reads (approx. 50 million sequenced fragments) per sample remained on average. The number of aligned reads surpassed the ENCODE recommendations by at least 30% (92 and 85 million on average in the young and old cohorts, respectively; [40]).

We found 61 differentially expressed genes after removing the hemoglobin-related genes. An unexpected observation was that the range of the fold change values of the significant hits was much lower than in the recent RNA-seq study on dogs [12], which was performed on tissue samples from the brain’s prefrontal cortex region. The log2 fold change was between −1 and 2.5 in the current study, whereas it was between −8.19 and 7.54 in our dog vs. wolf study [41] and between −8.31 and 4.46 when we compared gene expression profiles in young and old dogs’ brain [12]. We performed a technical replicate of the DESeq2 analysis with the edgeR R package—another popular tool for differential gene expression analysis—which confirmed our results that there are no significantly differentially expressed genes. Given the low number of differentially expressed genes (n = 61), it is not surprising that the gene ontology (GO) analysis identified only “response to bacterium” as a significant GO term. This finding suggests a potential involvement of the immune system in aging-related gene expression changes. It is possible that the observed difference in gene expression reflects dysregulated immune function in older animals, which could be a characteristic of aging, as immune senescence is known to impact aging in many species. However, the hypothesis that immune dysfunction specifically drives these changes in the older cohort remains speculative. We cannot definitively verify this connection or draw concrete conclusions without further experiments, such as analyzing immune system biomarkers and inflammatory cytokine profiles or assessing immune cell composition and activity in aged dogs. Thus, while this observation is intriguing, more targeted research is needed to confirm the role of immune dysregulation in the aging process of dogs.

Hemoglobin-related reads were removed after alignment to the reference genome, following the recommendations of Harrington et al. [14]. The removal had an uneven impact on the analyzed samples, with 24–75% of the reads being excluded. This led to a disproportional reduction in read counts across the samples. However, several indicators suggest that this did not significantly affect our analysis. First, the different techniques used to analyze the raw data—such as multidimensional scaling, principal component analysis, and sample distance calculations—yielded consistent results with each other and with the differential gene expression analysis, increasing confidence in the validity of our findings. Second, the MA plot (Figure 2) did not reveal a significant difference in read counts between the two age cohorts, suggesting that no systematic bias was introduced by the removal of hemoglobin-related reads. Finally, when the same dataset was analyzed in a different context (investigating differentially expressed genes between companion dogs and wolves [41], with hemoglobin-related reads similarly excluded), significant differential expression was observed, and 90% of the differentially expressed genes from previous studies were identified, along with additional genes (n = 1396). These results indicated that the sequencing depth was adequate for differential gene expression analysis, even after removing the hemoglobin-related reads.

When we compared the hemoglobin-related reads between our dataset and that of Yang et al. [15], similar patterns were observed. This comparison included total read counts, the proportion of all reads, and the number and proportion of secondary alignments (some information about the data from Yang et al. [15] can be found in Table S3, while our own data are presented in Table 3). Therefore, our samples were not extreme in this regard. A large proportion of the reads aligned with hemoglobin genes in both datasets, with average hemoglobin-related reads being 48% in our study and 50% in the dataset of Yang et al. [15]. The variance in the number of hemoglobin genes between the samples was similarly high in both datasets. Hemoglobin genes are paralogous, with 30–50% cDNA sequence identity, according to Ensembl [30]. This high sequence identity explains the significant increase in the proportion of multimapping reads, which were more than 10 times higher on average (Table 3). After removing the hemoglobin-related reads, the proportion of secondary alignments decreased to a normal level of 3% in all datasets. A similarly high proportion of globin reads (81%) was reported in a human study without globin depletion [42].

Previously, Kim et al. [24] implemented a similar study, which also investigated gene expression differences as a function of age and identified 154 significantly differentially expressed genes. However, it is worth noting that Kim et al. did not adjust *p*-values for multiple testing, which may have increased the type I error rate in their hypothesis testing. As stated in the materials and methods section of their manuscript, they considered genes with a *p*-value < 0.05 and |log2FC| > 1 as statistically significant, without correction for multiple comparisons. If appropriate measures had been taken to control the type I error rate at the conventional 5% level, their results might not have remained significant (see Table S2 of Kim et al. [24]). This aligns with our current findings, where we found fewer significant changes.

More recently, Zeng et al. [25] found a similar number of significantly differentially expressed genes than us, independently from us on a larger sample. Their sample included 30 individuals from another breed (Belgian malinois) and treated time as a continuous variable in contrast to our discrete age groups. Comparing the two sets of differentially expressed genes, 67% of them overlapped. This proportion is even higher (84%) if only known genes (i.e., genes with proper gene names) are considered. In total, 83% of the overlapping genes changed in the same direction in both studies (note: for this comparison, the changes from 1–2 years old to 8–9 years old were assessed in the dataset of Zeng et al., as that age range imitated the best our discrete age groups; see also Table S4). These independent studies increase the reliability of the obtained results and indicate that the overlapping genes might be of greater interest in future investigations of age-related blood biomarkers.

In contrast to our results, Charruau et al. [23] identified 625 differentially expressed genes between three young and two old wolves, with 214 genes upregulated and 411 genes downregulated. They also reported several enriched gene ontology (GO) terms among these genes, many of which “regulation of metabolic process”, “RNA metabolic process”, “chromatin modification”, and “immune response”, have been previously identified in human studies. One key difference between Charruau et al.’s study and ours is that they collected samples from 27 wolves living in Yellowstone National Park, where the environmental conditions are likely to be more similar across the animals, whereas our companion dogs lived in different households, potentially exposing them to a wider range of environmental factors.

There were also notable differences in the experimental design and methodology. For instance, Charruau et al. included younger individuals (<1 year old) in their study, while our study focused exclusively on two distinct age cohorts, excluding the juvenile stage. They also explored correlations across a broader range of age groups, whereas our study specifically compared older and younger adults.

While these methodological differences may explain some of the variation in findings, biological factors could also play a role. For example, environmental factors such as physical activity and diet can influence blood cell gene expression [43]. In our study, one potential confounding factor that we were able to exclude was breed, as we sampled only a single breed (border collie) to minimize within-group variance. However, environmental factors may have acted as confounding variables, preventing the identification of differentially expressed genes. Conversely, the more homogeneous environmental conditions for the wolves in Charruau et al.’s study could have reduced such confounding effects, potentially making it easier to detect age-related gene expression changes. This suggests that environmental influences, in addition to experimental design, could significantly impact the results of transcriptomic studies in different species.

The differential transcript usage analysis complemented our differential gene expression analysis by detecting changes at the transcript level rather than the gene level. Although the results at the transcript level were more convincing, the limited number of associated genes (n = 30) restricted further analysis, such as gene ontology analysis. Nevertheless, these findings suggest that investigating age-related changes in the blood transcriptome could be a promising avenue for future research. The list of differentially expressed genes, along with all relevant parameters, is available in Table S2.

## 5. Conclusions

In conclusion, although we did not identify any genes that were significantly differentially expressed between young and old companion dogs in whole blood samples, we did observe differential transcript usage for a limited number of genes, which holds promise for future research. Blood, as a tissue, is in contact with all other tissues in both animals and humans, meaning that changes in blood composition and gene expression levels can provide valuable information for a wide range of diseases and phenotypes, including healthy aging. By characterizing gene expression changes during normal aging, scientists and healthcare practitioners can more easily detect disease-related alterations.

Based on our findings, we suggest that future studies simplify the studied populations to reduce confounding factors such as diet, physical activity, and healthcare history, which could influence gene expression and to remove red blood cells prior to sequencing or, if not feasible, perform in silico removal of hemoglobin-related reads to improve data quality and reduce the potential impact of hemoglobin on analysis.

## Figures and Tables

**Table 1:** Summary of the analyzed samples.

ID	Age (Years)	Breed	Sex	Neutered	RNA Concentration (ng/µL)
CL_y1	1	Border collie	Male	Yes	56.4
CL_y2	1	Border collie	Female	No	38.6
CL_y3	3	Border collie	Female	No	67.0
CL_y4	1	Border collie	Female	Yes	42.2
CL_y5	1	Border collie	Female	No	36.8
CL_o1	11	Border collie	Female	Yes	53.0
CL_o2	10	Border collie	Male	No	38.4
CL_o3	15	Border collie	Female	Yes	31.8
CL_o4	14	Border collie	Male	No	43.2
CL_o5	10	Border collie	Female	Yes	50.8

**Table 2:** Hemoglobin-related genes identified by Harrington et al. [14] and their corresponding canine orthologs, along with associated genomic information. The dashed line distinguishes between active and archived canine genes in the Ensembl annotation database.

Chromosome	Gene Start	Gene End	Strand	Dog Gene ID	Human Gene ID	Human Gene Abbreviation	Human Gene Name	Gene Type	Ortholog Type	Dog Gene’s Status
6	40,324,263	40,326,351	Reverse	ENSCAFG00000032615	ENSG00000188536	HBA2	hemoglobin subunit alpha 2	Protein coding	Many to many	Active
6	40,324,263	40,326,351	Reverse	ENSCAFG00000032615	ENSG00000206172	HBA1	hemoglobin subunit alpha 1	Protein coding	Many to many	Active
6	40,330,683	40,332,438	Reverse	ENSCAFG00000029224	ENSG00000206177	HBM	hemoglobin subunit mu	Protein coding	One to one	Active
6	40,342,562	40,343,504	Reverse	ENSCAFG00000028569	ENSG00000130656	HBZ	hemoglobin subunit zeta	Protein coding	One to many	Active
21	28,181,347	28,205,138	Reverse	ENSCAFG00000030286	ENSG00000223609	HBD	hemoglobin subunit delta	Protein coding	Many to many	Active
21	28,181,347	28,205,138	Reverse	ENSCAFG00000030286	ENSG00000244734	HBB	hemoglobin subunit beta	Protein coding	Many to many	Active
6	40,326,459	40,329,857	Reverse	ENSCAFG00000029904	ENSG00000188536	HBA2	hemoglobin subunit alpha 2	Protein coding	Many to many	Archived
6	40,326,459	40,329,857	Reverse	ENSCAFG00000029904	ENSG00000206172	HBA1	hemoglobin subunit alpha 1	Protein coding	Many to many	Archived
6	40,331,793	40,332,696	Reverse	ENSCAFG00000031055	ENSG00000130656	HBZ	hemoglobin subunit zeta	Protein coding	One to many	Archived
21	28,179,119	28,180,299	Reverse	ENSCAFG00000029518	ENSG00000223609	HBD	hemoglobin subunit delta	Protein coding	Many to many	Archived
21	28,179,119	28,180,299	Reverse	ENSCAFG00000029518	ENSG00000244734	HBB	hemoglobin subunit beta	Protein coding	Many to many	Archived
21	28,193,272	28,194,670	Reverse	ENSCAFG00000024181	ENSG00000196565	HBG2	hemoglobin subunit gamma 2	Protein coding	One to many	Archived
21	28,193,272	28,194,670	Reverse	ENSCAFG00000024181	ENSG00000213934	HBG1	hemoglobin subunit gamma 1	Protein coding	One to many	Archived

**Table 3:** Sequencing and alignment statistics of the 10 sequenced samples. The dashed line separates the individual results from the summary data per group.

Sample ID	Number of Reads		Number of Aligned Reads		Number and Proportion of Hemoglobin Reads		Secondary Alignments with Hemoglobin Genes		Secondary Alignments Without Hemoglobin Genes	
	Sequenced	After Quality Control	N	%	N	%	N	%	N	%
CL_y1	139,574,092	99,781,996	87,552,180	87.7	64,061,880	73.2	100,056,226	100.3	2,175,945	2.2
CL_y2	145,101,716	114,231,400	98,754,640	86.5	24,426,835	24.7	33,712,226	29.5	5,736,365	5.0
CL_y3	211,298,362	137,241,346	120,949,359	88.1	91,142,725	75.4	174,121,970	126.9	2,585,114	1.9
CL_y4	115,495,360	102,506,414	87,090,986	85.0	20,523,206	23.6	36,225,285	35.3	4,687,901	4.6
CL_y5	100,769,724	76,546,858	66,152,258	86.4	35,704,823	54.0	51,500,272	67.3	2,617,499	3.4
CL_o1	132,309,048	100,814,174	86,252,499	85.6	38,902,610	45.1	75,151,359	74.5	4,017,804	4.0
CL_o2	115,616,142	93,060,894	79,023,663	84.9	37,103,408	47.0	57,135,456	61.4	3,463,513	3.7
CL_o3	120,024,014	91,619,710	80,186,573	87.5	29,403,124	36.7	31,520,157	34.4	3,990,157	4.4
CL_o4	124,942,900	103,191,914	88,892,129	86.1	30,694,317	34.5	54,958,699	53.3	3,720,187	3.6
CL_o5	148,054,394	103,245,778	90,434,886	87.6	58,026,387	64.2	111,519,462	108.0	2,956,990	2.9
young average	142,447,851	106,061,603	92,099,885	86.7	47,171,894	50.2	79,123,196	71.9	3,560,565	3.4
old average	128,189,300	98,386,494	84,957,950	86.4	38,825,969	45.5	66,057,027	66.3	3,629,730	3.7

## Data Availability

The genetic data generated and analyzed in this study can be found in the [American] National Center for Biotechnology Information’s Sequence Read Archive under BioProject ID: PRJNA823683. The laboratory blood test data are available in Table S1. All log files and scripts used for data analysis are publicly available on Science Data Bank (https://www.scidb.cn (uploaded on 21 August 2023)) with the following DOI accession number: 10.57760/sciencedb.09567. In case of questions, please contact the corresponding author.

## Supplementary Materials

The following supporting information can be downloaded at: https://www.mdpi.com/article/10.3390/vetsci12020086/s1, Figure S1. Raw read count transformations with three transformation methods; Figure S2. Schematic presentation of the applied analysis pipeline; Table S1. Blood test results of the sequenced animals. Table S2. Basic information on the statistically significant differentially expressed genes; Table S3. Sequencing and alignment statistics of the 5 samples published by Yang et al. (2018) [15]; Table S4. Comparison of the results of Zeng et al. (2024) [25] and the current study; Figure S3. Results of a standard principal component analysis of the read counts of the 10 samples; Figure S4. Sample distances calculated from the rlog-transformed raw read counts of all expressed genes; Figure S5. Correlations (A) between the initial RNA concentration levels and raw number of sequenced fragments before hemoglobin depletion and (B) between RNA concentration levels and the number of aligned reads, after in silico hemoglobin depletion.
