# Supplementary material for: Differential Gene Expression Analysis of Whole Blood Transcriptome Between Young and Old Border Collie Dogs
Source: Vet Sci. 2025 Jan 23;12(2):86. doi: 10.3390/vetsci12020086 (PMC11860333; doi:10.3390/vetsci12020086)
Supplement: Supplementary file 1 [file vetsci-12-00086-s001.zip › vetsci-3375433-supplementary.pdf]

Supplementary Material

Differential Gene Expression Analysis of Whole Blood Transcriptome Between Young and Old Border Collie Dogs

Dávid Jónás [1,2,†], Kitti Tátrai [1,3,†], Zsófia Rékasi [1], Balázs Egyed [3,*] and Eniko Kubinyi [1,2,4,*]

[1] Department of Ethology, ELTE Eötvös Loránd University, Pázmány Péter sétány 1/c, 1117 Budapest, Hungary; jonas.david@ttk.elte.hu (D.J.); tatraikitti@student.elte.hu (K.T.); rekasizsofi@student.elte.hu (Z.R.)
[2] MTA-ELTE Lendület "Momentum" Companion Animal Research Group, Pázmány Péter sétány 1/c, 1117
Budapest, Hungary
[3] Department of Genetics, ELTE Eötvös Loránd University, Pázmány Péter sétány 1/c, 1117 Budapest, Hungary
[4] ELTE NAP Canine Brain Research Group, Pázmány Péter sétány 1/c, 1117 Budapest, Hungary
* Correspondence: egyed.balazs@ttk.elte.hu (B.E.); eniko.kubinyi@ttk.elte.hu (E.K.)
† These authors contributed equally to this work.

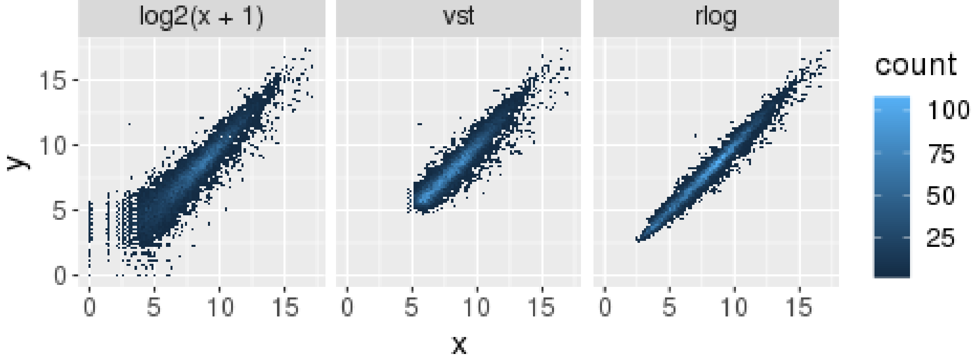

Figure S1. Raw read count transformations with three transformation methods: 1) the logarithm (base: 2) transformation; 2) the variance stabilizing transformation (vst) and 3) the regularized logarithm transformation. Read counts are shown for the first 2 individuals of our dataset.

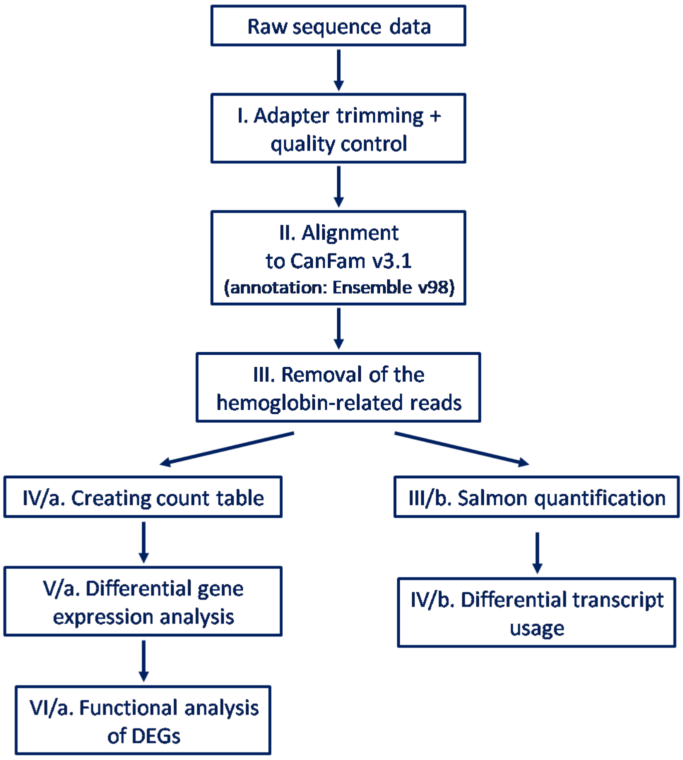

Figure S2. Schematic presentation of the applied analysis pipeline.

Table S1. Blood test results of the sequenced animals. The red color indicates a deviation from the reference range.

						AST (GOT)		ALT (GPT)	
**ID**	**Age (years)**	**Sex**	**Neutered**	**Pedigree**	**Line**	**Value**	**Ref**	**Value**	**Ref**
CL_y1	1	M	Y	Y	Herding	22 IU/l	10-50 IU/l	33 IU/l	10-60 IU/l
CL_y2	1	F	N	Y	Show	27 IU/l	10-50 IU/l	23 IU/l	10-60 IU/l
CL_y3	3	F	N	N	Mix	25 IU/l	10-50 IU/l	33 IU/l	10-60 IU/l
CL_y4	1	F	Y	N	Mix	49 IU/l	10-50 IU/l	47 IU/l	10-60 IU/l
CL_y5	1	F	N	Y	Show	32 IU/l	10-50 IU/l	42 IU/l	10-60 IU/l
CL_o1	11	F	Y	Y	Show	19 IU/l	10-50 IU/l	41 IU/l	10-60 IU/l
CL_o2	10	M	N	Y	Show	34 IU/l	10-50 IU/l	39 IU/l	10-60 IU/l
CL_o3	15	F	Y	?	Show	28 IU/l	10-50 IU/l	25 IU/l	10-60 IU/l
CL_o4	14	M	N	Y	Show	26 IU/l	10-50 IU/l	**87 IU/l**	10-60 IU/l
CL_o5	10	F	Y	Y	Show	43 IU/l	10-50 IU/l	38 IU/l	10-60 IU/l

	ALKP		GGT		Total bilirubin		Direct Bilirubin	
**ID**	**Value**	**Ref**	**Value**	**Ref**	**Value**	**Ref**	**Value**	**Ref**
CL_y1	179 IU/l	20-300 IU/l	1 IU/l	< 10 IU/l	< 10 umol/l	< 18 umol/l	<5 umol/l	< 5 umol/l
CL_y2	76 IU/l	20-300 IU/l	2 IU/l	< 10 IU/l	< 10 umol/l	< 18 umol/l	<5 umol/l	< 5 umol/l
CL_y3	-	20-300 IU/l	3 IU/l	< 10 IU/l	< 10 umol/l	< 18 umol/l	<5 umol/l	< 5 umol/l
CL_y4	79 IU/l	20-300 IU/l	2 IU/l	< 10 IU/l	< 10 umol/l	< 18 umol/l	<5 umol/l	< 5 umol/l
CL_y5	-	20-300 IU/l	-	< 10 IU/l	< 10 umol/l	< 18 umol/l	<5 umol/l	< 5 umol/l
CL_o1	-	20-300 IU/l	5 IU/l	< 10 IU/l	< 10 umol/l	< 18 umol/l	<5 umol/l	< 5 umol/l
CL_o2	-	20-300 IU/l	5 IU/l	< 10 IU/l	< 10 umol/l	< 18 umol/l	<5 umol/l	< 5 umol/l
CL_o3	162 IU/l	20-300 IU/l	4 IU/l	< 10 IU/l	< 10 umol/l	< 18 umol/l	<5 umol/l	< 5 umol/l
CL_o4	144 IU/l	20-300 IU/l	10 IU/l	< 10 IU/l	< 10 umol/l	< 18 umol/l	<5 umol/l	< 5 umol/l
CL_o5	238 IU/l	20-300 IU/l	4 IU/l	< 10 IU/l	< 10 umol/l	< 18 umol/l	<5 umol/l	< 5 umol/l

	Total protein		Albumin		Albumin/Globulin ratio		Glucose	
**ID**	**Value**	**Ref**	**Value**	**Ref**	**Value**	**Ref**	**Value**	**Ref**
CL_y1	61.9 g/l	60-80 g/l	31.4 g/l	25-45 g/l	1.0	0.5-1.5	5.3 mmol/l	3.8-6.0 mmol/l
CL_y2	66.0 g/l	60-80 g/l	26.2 g/l	25-45 g/l	0.7	0.5-1.5	**3.1 mmol/l**	3.8-6.0 mmol/l
CL_y3	**81.6 g/l**	60-80 g/l	28.2 g/l	25-45 g/l	0.5	0.5-1.5	**6.7 mmol/l**	3.8-6.0 mmol/l
CL_y4	61.4 g/l	60-80 g/l	30.6 g/l	25-45 g/l	1.0	0.5-1.5	4.1 mmol/l	3.8-6.0 mmol/l
CL_y5	65.7 g/l	60-80 g/l	31.0 g/l	25-45 g/l	0.9	0.5-1.5	5.2 mmol/l	3.8-6.0 mmol/l
CL_o1	72.8 g/l	60-80 g/l	29.3 g/l	25-45 g/l	0.7	0.5-1.5	5.3 mmol/l	3.8-6.0 mmol/l
CL_o2	**81.6 g/l**	60-80 g/l	33.3 g/l	25-45 g/l	0.7	0.5-1.5	4.1 mmol/l	3.8-6.0 mmol/l
CL_o3	75.0 g/l	60-80 g/l	26.5 g/l	25-45 g/l	0.5	0.5-1.5	4.6 mmol/l	3.8-6.0 mmol/l
CL_o4	64.5 g/l	60-80 g/l	28.5 g/l	25-45 g/l	0.8	0.5-1.5	5.3 mmol/l	3.8-6.0 mmol/l
CL_o5	**83.2 g/l**	60-80 g/l	28.9 g/l	25-45 g/l	0.5	0.5-1.5	**2.2 mmol/l**	3.8-6.0 mmol/l

	Fructose amin		Total cholesterine		Triglicerid		Karbamid	
ID	Value	Ref	Value	Ref	Value	Ref	Value	Ref
CL_y1	264 umol/l	100-344 umol/l	6.1 mmol/l	2.9-10.4 mmol/l	0.63 mmol/l	0.34-1.59 mmol/l	9.5 mmol/l	2.9-10.4 mmol/l
CL_y2	165 umol/l	100-344 umol/l	6.2 mmol/l	2.9-10.4 mmol/l	1.16 mmol/l	0.34-1.59 mmol/l	5.8 mmol/l	2.9-10.4 mmol/l
CL_y3	269 umol/l	100-344 umol/l	4.9 mmol/l	2.9-10.4 mmol/l	0.5 mmol/l	0.34-1.59 mmol/l	3 mmol/l	2.9-10.4 mmol/l
CL_y4	247 umol/l	100-344 umol/l	6.2 mmol/l	2.9-10.4 mmol/l	**0.32 mmol/l**	0.34-1.59 mmol/l	9.8 mmol/l	2.9-10.4 mmol/l
CL_y5	215 umol/l	100-344 umol/l	3.7 mmol/l	2.9-10.4 mmol/l	0.34 mmol/l	0.34-1.59 mmol/l	7.1 mmol/l	2.9-10.4 mmol/l
CL_o1	212 umol/l	100-344 umol/l	6.8 mmol/l	2.9-10.4 mmol/l	1.09 mmol/l	0.34-1.59 mmol/l	6.7 mmol/l	2.9-10.4 mmol/l
CL_o2	327 umol/l	100-344 umol/l	6.8 mmol/l	2.9-10.4 mmol/l	0.67 mmol/l	0.34-1.59 mmol/l	**13.5 mmol/l**	2.9-10.4 mmol/l
CL_o3	308 umol/l	100-344 umol/l	6.7 mmol/l	2.9-10.4 mmol/l	**2.46 mmol/l**	0.34-1.59 mmol/l	**13.5 mmol/l**	2.9-10.4 mmol/l
CL_o4	296 umol/l	100-344 umol/l	9.1 mmol/l	2.9-10.4 mmol/l	1.31 mmol/l	0.34-1.59 mmol/l	**10.6 mmol/l**	2.9-10.4 mmol/l
CL_o5	240 umol/l	100-344 umol/l	6.1 mmol/l	2.9-10.4 mmol/l	0.74 mmol/l	0.34-1.59 mmol/l	6.7 mmol/l	2.9-10.4 mmol/l

	Creatinin		P		Na		K	
ID	Value	Ref	Value	Ref	Value	Ref	Value	Ref
CL_y1	128 umol/l	40-140 umol/l	1.5 mmol/l	0.8-2.0 mmol/l	150.2 mmol/l	135-155 mmol/l	4.7 mmol/l	3.5-5.4 mmol/l
CL_y2	90 umol/l	40-140 umol/l	1.5 mmol/l	0.8-2.0 mmol/l	145.7 mmol/l	135-155 mmol/l	5.3 mmol/l	3.5-5.4 mmol/l
CL_y3	92 umol/l	40-140 umol/l	-	0.8-2.0 mmol/l	-	135-155 mmol/l	-	3.5-5.4 mmol/l
CL_y4	81 umol/l	40-140 umol/l	1.5 mmol/l	0.8-2.0 mmol/l	151.9 mmol/l	135-155 mmol/l	4.4 mmol/l	3.5-5.4 mmol/l
CL_y5	107 umol/l	40-140 umol/l	-	0.8-2.0 mmol/l	-	135-155 mmol/l	-	3.5-5.4 mmol/l
CL_o1	95 umol/l	40-140 umol/l	0.8 mmol/l	0.8-2.0 mmol/l	-	135-155 mmol/l	-	3.5-5.4 mmol/l
CL_o2	**144 umol/l**	40-140 umol/l	0.8 mmol/l	0.8-2.0 mmol/l	-	135-155 mmol/l	-	3.5-5.4 mmol/l
CL_o3	130 umol/l	40-140 umol/l	1.4 mmol/l	0.8-2.0 mmol/l	152.8 mmol/l	135-155 mmol/l	4.9 mmol/l	3.5-5.4 mmol/l
CL_o4	122 umol/l	40-140 umol/l	1.2 mmol/l	0.8-2.0 mmol/l	148.1 mmol/l	135-155 mmol/l	4.8 mmol/l	3.5-5.4 mmol/l
CL_o5	95 umol/l	40-140 umol/l	1.3 mmol/l	0.8-2.0 mmol/l	146.7 mmol/l	135-155 mmol/l	5.3 mmol/l	3.5-5.4 mmol/l

	Na/K ratio		Ca		Mg		Fe	
ID	Value	Ref	Value	Ref	Value	Ref	Value	Ref
CL_y1	32	27-40	2.1 mmol/l	2.0-3.0 mmol/l	0.8 mmol/l	0.66-1.2 mmol/l	21.2 umol/l	5.9-45 umol/l
CL_y2	27.5	27-40	2.5 mmol/l	2.0-3.0 mmol/l	0.89 mmol/l	0.66-1.2 mmol/l	18.3 umol/l	5.9-45 umol/l
CL_y3	-	27-40	-	2.0-3.0 mmol/l	-	0.66-1.2 mmol/l	-	5.9-45 umol/l
CL_y4	34.5	27-40	2.4 mmol/l	2.0-3.0 mmol/l	0.75 mmol/l	0.66-1.2 mmol/l	23.1 umol/l	5.9-45 umol/l
CL_y5	-	27-40	-	2.0-3.0 mmol/l	-	0.66-1.2 mmol/l	-	5.9-45 umol/l
CL_o1	-	27-40	-	2.0-3.0 mmol/l	-	0.66-1.2 mmol/l	-	5.9-45 umol/l
CL_o2	-	27-40	-	2.0-3.0 mmol/l	-	0.66-1.2 mmol/l	-	5.9-45 umol/l
CL_o3	31.2	27-40	2.3 mmol/l	2.0-3.0 mmol/l	0.76 mmol/l	0.66-1.2 mmol/l	10.2 umol/l	5.9-45 umol/l
CL_o4	30.9	27-40	2.2 mmol/l	2.0-3.0 mmol/l	**1.39 mmol/l**	0.66-1.2 mmol/l	22.7 umol/l	5.9-45 umol/l
CL_o5	27.7	27-40	2.1 mmol/l	2.0-3.0 mmol/l	0.8 mmol/l	0.66-1.2 mmol/l	12.2 umol/l	5.9-45 umol/l

	CK		LDH	
**ID**	**Value**	**Ref**	**Value**	**Ref**
CL_y1	114 IU/l	30-374 IU/l	123 IU/l	20-280 IU/l
CL_y2	173 IU/l	30-374 IU/l	**344 IU/l**	20-280 IU/l
CL_y3	193 IU/l	30-374 IU/l	157 IU/l	20-280 IU/l
CL_y4	328 IU/l	30-374 IU/l	222 IU/l	20-280 IU/l
CL_y5	225 IU/l	30-374 IU/l	279 IU/l	20-280 IU/l
CL_o1	89 IU/l	30-374 IU/l	86 IU/l	20-280 IU/l
CL_o2	109 IU/l	30-374 IU/l	120 IU/l	20-280 IU/l
CL_o3	134 IU/l	30-374 IU/l	88 IU/l	20-280 IU/l
CL_o4	105 IU/l	30-374 IU/l	134 IU/l	20-280 IU/l
CL_o5	**688 IU/l**	30-374 IU/l	**403 IU/l**	20-280 IU/l

Table S2. Basic information on the statistically significant differentially expressed genes.

chr[1]	Start	End	Strand	Gene_ID	Gene name	Gene version[2]	Gene biotype	Gene source
MT	1090	2670	+	ENSCAFG00000022711	NA	1	Mt_rRNA	RefSeq
MT	5211	5279	-	ENSCAFG00000022721	NA	1	Mt_tRNA	RefSeq
1	107705575	107720876	-	ENSCAFG00000003981	SULT2B1	4	protein_coding	ensembl
1	109614436	109618242	+	ENSCAFG00000029461	PGLYRP1	2	protein_coding	ensembl
4	22916095	22964035	+	ENSCAFG00000032469	DDIT4	2	protein_coding	ensembl
5	29035819	29046808	+	ENSCAFG00000023335	MMP8	3	protein_coding	ensembl
5	61773459	61793873	+	ENSCAFG00000046831	NA	1	lncRNA	ensembl
6	59158854	59169646	+	ENSCAFG00000020200	GBP6	6	protein_coding	ensembl
8	45438238	46009049	+	ENSCAFG00000016763	RGS6	5	protein_coding	ensembl
9	14998012	15005546	+	ENSCAFG00000011099	AMZ2	4	protein_coding	ensembl
9	22068774	22090656	+	ENSCAFG00000030300	CCR7	2	protein_coding	ensembl
12	14428405	14687241	-	ENSCAFG00000002023	RCAN2	5	protein_coding	ensembl
13	34579125	35115494	-	ENSCAFG00000001181	TRAPPC9	4	protein_coding	ensembl
14	59951215	60000212	-	ENSCAFG00000003532	AASS	5	protein_coding	ensembl
16	278956	431704	+	ENSCAFG00000012301	TNS3	4	protein_coding	ensembl
17	37019328	37024458	-	ENSCAFG00000007249	IL1B	4	protein_coding	ensembl
24	42602594	42656209	-	ENSCAFG00000031382	PMEPA1	2	protein_coding	ensembl
27	9782804	10235028	-	ENSCAFG00000009609	TMEM117	5	protein_coding	ensembl
32	11353952	11362069	+	ENSCAFG00000009569	SPP1	5	protein_coding	ensembl
33	22904148	22920956	-	ENSCAFG00000010905	B4GALT4	4	protein_coding	ensembl
35	6508513	6573761	+	ENSCAFG00000039445	NA	2	lncRNA	ensembl
37	2414439	2635431	-	ENSCAFG00000010115	TMEFF2	4	protein_coding	ensembl
38	523480	720195	+	ENSCAFG00000009614	SOX13	4	protein_coding	ensembl
4	60559215	60569533	-	ENSCAFG00000018379	GZMK	4	protein_coding	ensembl
5	27863590	28089936	+	ENSCAFG00000032342	PDGFD	2	protein_coding	ensembl
7	6225187	6236918	+	ENSCAFG00000025093	C4BPB	4	protein_coding	ensembl
9	134999	230183	+	ENSCAFG00000006048	CCDC57	5	protein_coding	ensembl
9	54760275	54796848	+	ENSCAFG00000020030	KYAT1	4	protein_coding	ensembl
10	23728997	23741349	+	ENSCAFG00000001053	POLR3H	4	protein_coding	ensembl
12	49106522	49460286	-	ENSCAFG00000003116	BACH2	6	protein_coding	ensembl
20	40790810	40792794	-	ENSCAFG00000012896	CAMP	3	protein_coding	ensembl
20	42294995	42297952	-	ENSCAFG00000013783	CCR5	2	protein_coding	ensembl
23	27418355	27530961	+	ENSCAFG00000024259	NA	4	protein_coding	ensembl
24	17569662	17572127	+	ENSCAFG00000006187	CENPB	5	protein_coding	ensembl
27	27777325	28023987	-	ENSCAFG00000012478	PLEKHA5	4	protein_coding	ensembl
27	37777488	37809739	-	ENSCAFG00000014011	NA	4	protein_coding	ensembl
28	33933604	34081090	-	ENSCAFG00000012752	FAM53B	4	protein_coding	ensembl
32	10125440	10328757	+	ENSCAFG00000009427	PTPN13	4	protein_coding	ensembl
33	17556909	17630259	+	ENSCAFG00000010582	BOC	4	protein_coding	ensembl
1	111693004	111698742	+	ENSCAFG00000004765	CD177	5	protein_coding	ensembl
3	65379531	65469252	+	ENSCAFG00000035173	NA	2	lncRNA	ensembl
4	263487	296916	-	ENSCAFG00000029179	ZNF25	2	protein_coding	ensembl
5	40466236	40474457	+	ENSCAFG00000018146	ALDH3A1	4	protein_coding	ensembl

chr[1]	Start	End	Strand	Gene_ID	Gene name	Gene version[2]	Gene biotype	Gene source
6	55347954	55525246	+	ENSCAFG00000020133	BCAR3	4	protein_coding	ensembl
7	37775126	38081733	+	ENSCAFG00000015982	CDC42BPA	5	protein_coding	ensembl
7	41382993	41419742	+	ENSCAFG00000016616	IQGAP3	4	protein_coding	ensembl
9	19380687	19386621	+	ENSCAFG00000048477	NA	1	lncRNA	ensembl
9	37618048	37622262	-	ENSCAFG00000032259	NA	2	protein_coding	ensembl
10	34660608	34661079	+	ENSCAFG00000001968	NA	3	protein_coding	ensembl
11	62450059	62535121	+	ENSCAFG00000002774	ZNF462	5	protein_coding	ensembl
12	11788720	11791258	-	ENSCAFG00000001842	CRIP3	4	protein_coding	ensembl
12	1193378	1197838	+	ENSCAFG00000031994	LY6G6D	2	protein_coding	ensembl
12	2563672	2568328	-	ENSCAFG00000000896	DLA-DOA	4	protein_coding	ensembl
12	7617591	7972842	+	ENSCAFG00000001518	DNAH8	5	protein_coding	ensembl
13	38518643	38814369	+	ENSCAFG00000001792	LIMCH1	5	protein_coding	ensembl
14	14032474	14250554	+	ENSCAFG00000001852	ADAM22	5	protein_coding	ensembl
17	37489766	37535252	+	ENSCAFG00000023369	NA	2	protein_coding	ensembl
19	25554966	26397896	-	ENSCAFG00000004689	CNTNAP5	4	protein_coding	ensembl
23	27596394	27721265	+	ENSCAFG00000006046	COL6A5	5	protein_coding	ensembl
25	43826173	43826904	+	ENSCAFG00000006486	NA	3	protein_coding	ensembl
35	20099772	20101154	+	ENSCAFG00000024883	SOX4	4	protein_coding	ensembl

1: chr – chromosome; 2: gene version – ENSEMBL gene identifier's version

Table S3. Sequencing and alignment statistics of the 5 samples published by Yang et al. (2018).

Sample ID	Number of sequenced reads	Number of reads after adapter trimming	Number of aligned reads		Number and proportion of hemoglobin reads		Secondary alignments with hemoglobin genes		Secondary alignments without hemoglobin genes	
			N	%	N	%	N	%	N	%
CL_d1	42,671,378	42,671,378	39,524,617	92.63	7,994,228	20.23	22,241,554	52.12	1,838,873	4.31
CL_d2	49,896,210	49,896,210	46,412,277	93.02	3,319,432	7.15	12,558,839	25.17	2,716,801	5.44
CL_w1	50,597,914	50,597,914	47,349,445	93.58	30,856,658	65.17	89,235,386	176.36	1,167,602	2.31
CL_w2	50,190,664	50,190,664	47,370,709	94.38	35,600,080	75.15	103,766,041	206.74	694,183	1.38
CL_w3	47,960,542	47,960,542	45,713,300	95.31	36,739,037	80.37	136,808,450	285.25	967,553	2.02
dog average	46,283,794	46,283,794	42,968,447	92.83	5,656,830	13.69	17,400,197	38.65	2,277,837	4.88
wolf average	49,583,040	49,583,040	46,811,151	94.42	34,398,592	73.48	109,936,626	222.78	943,113	1.90

Table S4. Comparison of the results of Zeng et al. (2024) and the current study. In total, 41 genes overlapped, out of which 34 (83%) changed in the same direction.

Gene ID	Gene name	Change with age (Zeng et al.)[1]	Fold change mark (current study)	Concordance between JD and Zeng
ENSCAFG00000022711	NA	NA	+	NA
ENSCAFG00000022721	NA	NA	+	NA
ENSCAFG00000046831	NA	NA	-	NA
ENSCAFG00000020200	GBP6	NA	+	NA
ENSCAFG00000039445	NA	NA	+	NA
ENSCAFG00000025093	C4BPB	NA	-	NA
ENSCAFG00000020030	KYAT1	NA	-	NA
ENSCAFG00000012896	CAMP	NA	+	NA
ENSCAFG00000024259	NA	NA	+	NA
ENSCAFG00000006187	CENPB	NA	-	NA
ENSCAFG00000014011	NA	NA	-	NA
ENSCAFG00000004765	CD177	NA	+	NA
ENSCAFG00000035173	NA	NA	+	NA
ENSCAFG00000029179	ZNF25	NA	+	NA
ENSCAFG00000048477	NA	NA	-	NA
ENSCAFG00000032259	NA	NA	+	NA
ENSCAFG00000001968	NA	NA	-	NA
ENSCAFG00000023369	NA	NA	-	NA
ENSCAFG00000004689	CNTNAP5	NA	+	NA
ENSCAFG00000006486	NA	NA	-	NA
ENSCAFG00000003981	SULT2B1	-	-	1
ENSCAFG00000029461	PGLYRP1	+	+	1
ENSCAFG00000032469	DDIT4	~Stable	-	0
ENSCAFG00000023335	MMP8	+	+	1
ENSCAFG00000016763	RGS6	+	+	1
ENSCAFG00000011099	AMZ2	+	+	1
ENSCAFG00000030300	CCR7	-	-	1
ENSCAFG00000002023	RCAN2	+	+	1
ENSCAFG00000001181	TRAPPC9	-	-	1
ENSCAFG00000003532	AASS	+	+	1
ENSCAFG00000012301	TNS3	-	-	1
ENSCAFG00000007249	IL1B	+	+	1
ENSCAFG00000031382	PMEPA1	-	-	1
ENSCAFG00000009609	TMEM117	+	+	1
ENSCAFG00000009569	SPP1	~Stable	+	0
ENSCAFG00000010905	B4GALT4	+	+	1
ENSCAFG00000010115	TMEFF2	~Stable	+	0
ENSCAFG00000009614	SOX13	-	-	1
ENSCAFG00000018379	GZMK	+	+	1

ENSCAFG00000032342	PDGFD	+	+	1
ENSCAFG00000006048	CCDC57	-	-	1
ENSCAFG00000001053	POLR3H	-	-	1
ENSCAFG00000003116	BACH2	-	-	1
ENSCAFG00000013783	CCR5	+	+	1
ENSCAFG00000012478	PLEKHA5	+	+	1
ENSCAFG00000012752	FAM53B	~Stable	-	0
ENSCAFG00000009427	PTPN13	+	+	1
ENSCAFG00000010582	BOC	-	-	1
ENSCAFG00000018146	ALDH3A1	-	-	1
ENSCAFG00000020133	BCAR3	-	-	1
ENSCAFG00000015982	CDC42BPA	+	-	0
ENSCAFG00000016616	IQGAP3	-	-	1
ENSCAFG00000002774	ZNF462	+	+	1
ENSCAFG00000001842	CRIP3	-	-	1
ENSCAFG00000031994	LY6G6D	~Stable	+	0
ENSCAFG00000000896	DLA-DOA	-	-	1
ENSCAFG00000001518	DNAH8	-	+	0
ENSCAFG00000001792	LIMCH1	-	-	1
ENSCAFG00000001852	ADAM22	-	-	1
ENSCAFG00000006046	COL6A5	+	+	1
ENSCAFG00000024883	SOX4	-	-	1

1: Changes from:young adult (1-2 years old dogs) to elderly adult (8-9 years old dogs) were considered, which age range are more similar to ours.

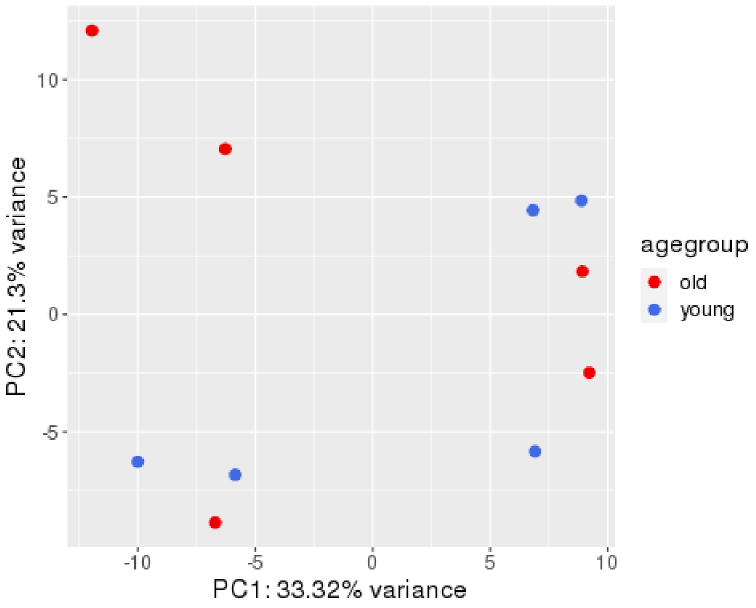

Figure S3. Results of a standard principal component analysis of the read counts of the 10 samples. The raw read counts were regularized logarithm transformed.

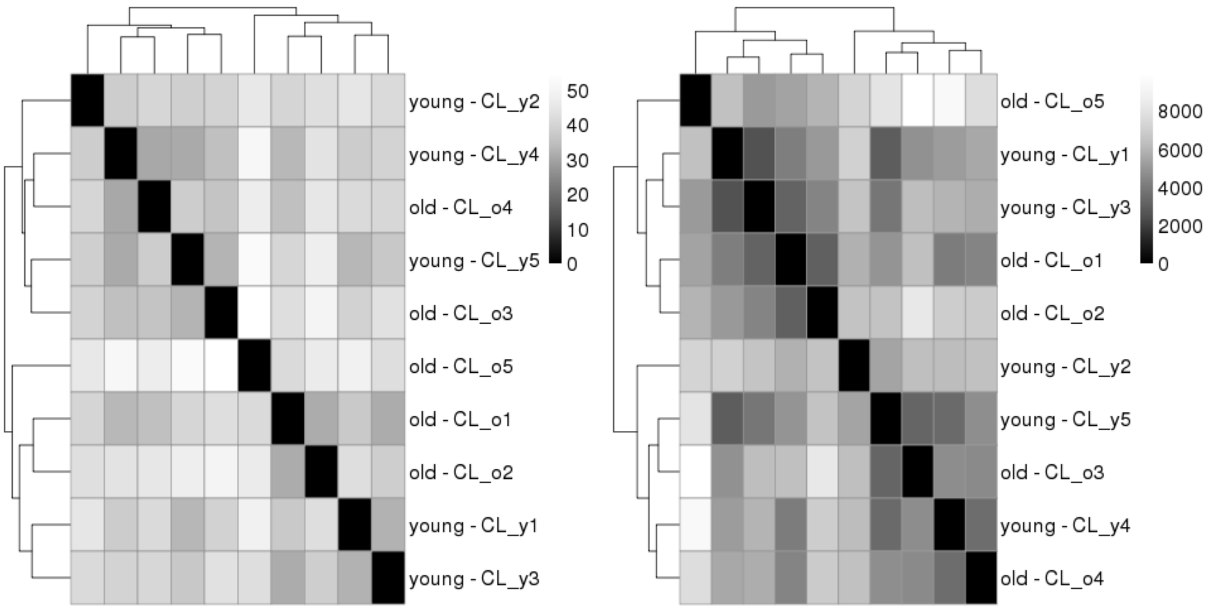

Figure S4. Sample distances calculated from the rlog-transformed raw read counts of all expressed genes. *Left:* Euclidean distances; *right:* Poisson distances.

Correlation between initial RNA concentrations and the raw number of sequenced reads

A strong correlation (r=0.81) was observed between the initial RNA concentration levels and the raw number of sequenced fragments in the samples (Fig. S5A). The correlation decreases to 59% if the outlier individual (CL_y3, with 67 ng/ul RNA concentration and 106 million fragments sequenced) is removed.
This correlation also decreased slightly after raw data quality control and alignment to the reference genome (r=0.72) but decreased greatly and turned from a positive to a negative correlation when the hemoglobin reads were removed from the analysis, after alignment (r=-0.51, Figure S5B). This latter value was much more similar to the same correlation we observed when we analyzed brain samples in a different study (r=-0.29; based on the dataset of Sándor, Jónás et al., 2022) and we conclude that the presence of the hemoglobin reads in the data also affects the correlation between observed RNA concentration values and the sequencing depth.

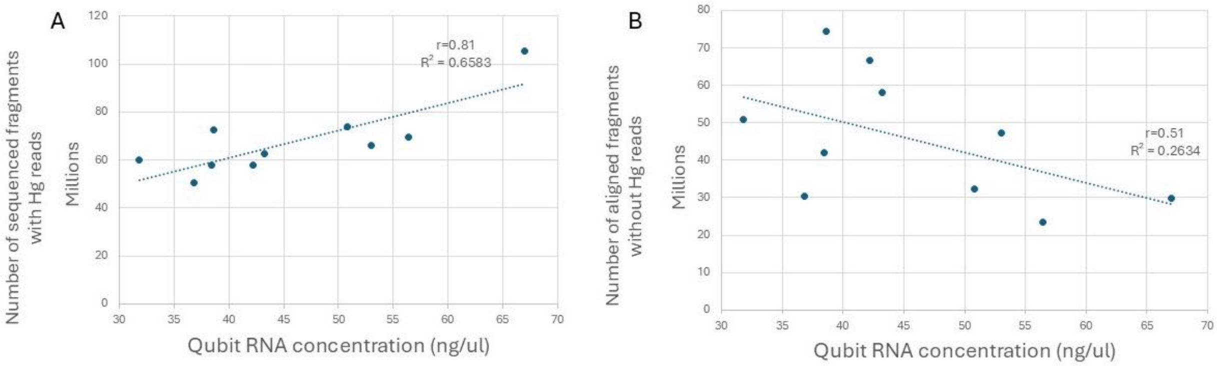

Figure S5. Correlations (A) between the initial RNA concentration levels and raw number of sequenced fragments before hemoglobin depletion and (B) between RNA concentration levels and the number of aligned reads, after *in silico* hemoglobin depletion.

References

Sándor, S.; Jónás, D.; Tátrai, K.; Czeibert, K.; Kubinyi, E. Poly(A)RNA sequencing reveals age-related differences in the prefrontal cortex of dogs. *Geroscience,* **2022**, 44, 1269-1293. https://doi.org/10.1007/s11357-022-00533-3.

Yang, X.; Zhang, H.; Shang, J.; Liu, G.; Xia, T.; Zhao, C.; Sun, G.; Dou, H. Comparative Analysis of the Blood Transcriptomes between Wolves and Dogs. *Anim. Genet.* **2018**, 49, 291–302. https://doi.org/10.1111/age.12675.

Zeng, M.; Zhou, T.; Li, Z.; Li, G.; Zhang, S.; Wang, L.; Huang, Q.G.; Li, J.D.; Samarawickrama, P.N.; He, Y.; et al. Transcriptomic and intervention evidence reveals domestic dogs as a promising model for anti-inflammatory investigation. *Aging Cell* **2024**, *23*, e14127, doi.org/10.1111/acel.14127.
